# IGT/LAZY genes are differentially influenced by light and required for light-induced change to organ angle

**DOI:** 10.1186/s12915-024-01813-4

**Published:** 2024-01-17

**Authors:** Jessica Marie Waite, Christopher Dardick

**Affiliations:** 1 United States Department of Agriculture (USDA) Appalachian Fruit Research Station, 2217 Wiltshire Road, Kearneysville, WV USA; 2 Present Address: USDA Tree Fruit Research Laboratory, 1104 N Western Avenue, Wenatchee, WA USA

**Keywords:** IGT family, *LAZY* family, Plant architecture, Gravitropic set-point angle, Branch angle, Root angle, Light signaling

## Abstract

**Background:**

Plants adjust their growth orientations primarily in response to light and gravity signals. Considering that the gravity vector is fixed and the angle of light incidence is constantly changing, plants must somehow integrate these signals to establish organ orientation, commonly referred to as gravitropic set-point angle (GSA). The IGT gene family contains known regulators of GSA, including the gene clades *LAZY*, *DEEPER ROOTING (DRO)*, and *TILLER ANGLE CONTROL (TAC)*.

**Results:**

Here, we investigated the influence of light on different aspects of GSA phenotypes in *LAZY* and *DRO* mutants, as well as the influence of known light signaling pathways on IGT gene expression. Phenotypic analysis revealed that *LAZY* and *DRO* genes are collectively required for changes in the angle of shoot branch tip and root growth in response to light. Single *lazy1* mutant branch tips turn upward in the absence of light and in low light, similar to wild-type, and mimic triple and quadruple IGT mutants in constant light and high-light conditions, while triple and quadruple IGT/*LAZY* mutants show little to no response to changing light regimes. Further, the expression of IGT/*LAZY* genes is differentially influenced by daylength, circadian clock, and light signaling.

**Conclusions:**

Collectively, the data show that differential expression of *LAZY* and *DRO* genes are required to enable plants to alter organ angles in response to light-mediated signals.

**Supplementary Information:**

The online version contains supplementary material available at 10.1186/s12915-024-01813-4.

## Background

Plant responses to light and gravity are crucial for development and survival. When environmental changes occur, such as light quality or the orientation of the plant with respect to gravity, the stimulus initiates a signaling cascade, relaying crucial information for the re-orientation of plant organs to ensure maximal access to light and soil resources. In this way, the overall shape, or architecture, of a plant is dependent on both light and gravity. A growing number of studies show that light and gravity pathways influence one another. In the hanging plant *Tradescantia*, treatment with different inhibitors of photosynthetic activity led to a change in the gravitropic set-point angle (GSA) of the branches [1], and in maize, coleoptiles grown in a rotating clinostat to reduce perceived gravity show an enhanced phototropic response [2]. The rice actin-binding protein RICE MORPHOLOGY DETERMINANT (RMD) highlights a link between light and gravity, acting to reorganize the actin cytoskeleton, allowing for proper sedimentation of amyloplasts, in the light and not dark [3]. In addition, recent studies performed in microgravity have begun to parse apart the individual influences of different light and gravity pathways on plant development and shape [4].

One aspect of plant architecture that is strongly influenced by both light and gravity is lateral organ orientation, or the angle at which organs such as branches, lateral roots, flower buds, and leaves grow. Optimizing lateral organ orientation can benefit crops in terms of space and resource use, both above and below ground [5–7]. Organ orientation with respect to gravity is also referred to as the gravitropic set-point angle (GSA) [8]. Work in Arabidopsis has shown that light conditions can alter lateral shoot GSA [9, 10] and seedling primary root responses to gravity [11], and leaf orientation is influenced by neighbor detection mechanisms using red/far-red light sensing in the leaf [12]. In rice, adventitious root GSA has also been shown to be influenced by light [13]. Taken together, the integration of light and gravity stimuli has a profound influence on GSA and the development of plant architecture.

The IGT gene family is central to the genetic control of lateral organ orientation. Named for a highly conserved “IGT” amino acid motif and alternatively referred to as the *LAZY* gene family, it is comprised of four distinct clades, three of which have been shown to play key roles: *LAZY*, *TILLER ANGLE CONTROL (TAC)*, and *DEEPER ROOTING (DRO)* [6, 14–21]. Recent studies have demonstrated that *LAZY* and *DRO* genes influence organ orientation downstream of amyloplast sedimentation and upstream of auxin localization [22–24]. In roots, the “anti-gravitropic” phenotype of a *lazy1 dro1 dro2 dro3* quadruple mutant requires functional statoliths, and in the shoot requires functional endodermal cells [25]. Mechanistic studies of DRO1 and DRO2 have shown that upon reorientation of roots, the proteins become polarly localized along the lower plasma membrane of the gravity sensing columella cells due to association with amyloplasts and recruit PRAF/RCC1-LIKE DOMAIN proteins, followed by subsequent changes in PIN localization [26, 27]. LAZY1 interaction with BREVIS RADIX LIKE 4 at the plasma membrane has also been shown recently to determine GSA and reorientation to gravity [28].

Recent studies have shown that both *TAC1* and *DRO1* are influenced by different light-related signals [10, 11]. *TAC1* is influenced by photosynthetic signals and contributes to branch and tiller orientation [10, 29]. DRO1 is activated by PHYTOCHROME INTERACTING FACTORS (PIFs) in the hypocotyl and by ELONGATED HYPOCOTYL 5 (HY5) in the root [11]. Earlier work in rice and maize compared *lazy1* mutant seedling responses in light and dark: rice *lazy1* shoot responses to reorientation were diminished in both dark and light, and maize *lazy1* mesocotyl elongation was accelerated in darkness and not light, which was inhibited by the addition of the auxin transport inhibitor, NPA [14, 18]. Maize *LAZY1* expression was also shown to be light- or circadian-mediated and was elevated in etiolated seedlings [18]. However, most studies on IGT mutants and the roles these genes play in gravitropism have been done in single-light conditions. Static phenotypic analysis of branch angles is often performed in either 16L:8D (long-day) or continuous light, while gravitropism assessments are often either in darkness (adult plants, hypocotyls, and roots) or red light or white light (roots) [20, 22, 23, 30, 31]. Here, we sought to understand the role of *LAZY* and *DRO* genes in light-induced changes to GSA, as well as how the expression of these genes is affected by light signaling pathways. We found that IGT genes are required for determining the angle of root and branch tip growth in response to light. We demonstrated through light treatments and photoreceptor mutant analysis that light-related signaling pathways differentially influenced *LAZY* and *DRO* gene expression. We conclude that *LAZY* and *DRO* genes are collectively required for light-induced changes to both shoot and root growth angles, and their expression is differentially regulated by light signals.

## Results

### Phenotypic responses of LAZY and DRO mutants to changes in light

**Fig. 1:**
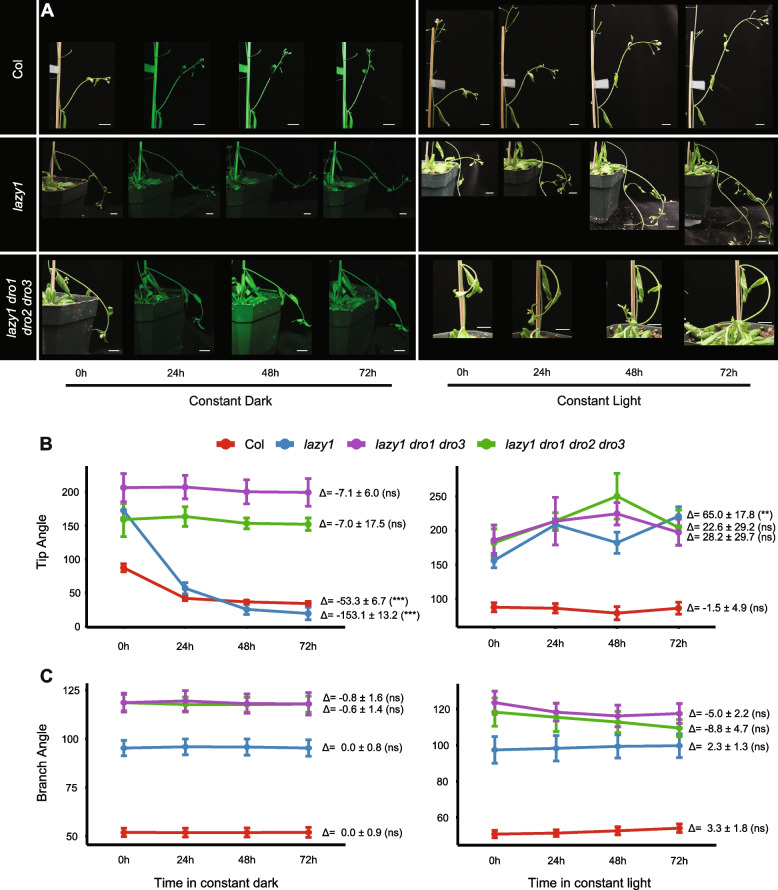
Loss of multiple *LAZY* and *DRO* genes confers branch angle insensitivity to changes in light. Time course of Col wild-type, *lazy1* mutant, and *lazy1 dro1 dro2 dro3* quadruple mutant grown in **A** constant dark (left) and constant light (right) conditions. White scale bars indicate 10 mm in length. **B** Quantified tip angles (tangent at 1 cm from the tip with respect to the stem) and **C** branch angles (angle to 2 cm from the branch point, with respect to the stem) for Col, *lazy1*, l*azy1 dro1 dro3* triple, and quadruple mutants in constant dark (left) and light (right) conditions. Error bars represent ± standard error of the mean at each time point, with *n* = 8 (Col), 10 (*lazy1*), 7 (triple), and 8 (quadruple) branches from four individual dark-grown plants, and *n* = 8 (Col), 8 (*lazy1*), 8 (triple), and 6 (quadruple) branches from four individual light-grown plants per time point. Angle change from 0 to 72 h for individual branches was averaged and reported with ± standard error of the mean to the right of each graph. Student’s *t*-test was used to determine the significant difference between 0- and 72-h time points and is reported in parentheses. *p < 0.05, **p < 0.01, ***p < 0.001

The reported connections between light and organ orientation [1, 9], and recent findings that both *TAC1* and *DRO1* are targets of different light signaling pathways [10, 11], led us to examine the relationship between light and organ angle in the context of IGT gene mutants. To measure the effects of *LAZY* and *DRO* gene loss on branch angle responses to daylength, we first transferred Arabidopsis wild-type (Col), *lazy1* single, *tac1 lazy1* double, *lazy1 dro1 dro3* triple, and *lazy1 dro1 dro2 dro3* quadruple mutants to continuous light and continuous dark regimes for 72 h [22, 23, 30]. Adult plants were grown in long-day conditions until they began initiating branches and reached ~ 15–22 cm in height, then were moved into light treatments. To track the dynamics of response to light and dark, individual branches were imaged every 24 h and the following angles were measured: the angle from the branch point to 2 cm down the branch, with respect to the main stem (referred to as branch angle hereafter) and the angle of tip growth, measured as the tangent at 1 cm from the branch tip with respect to the stem (referred to hereafter as tip angle) (Additional file 1: Fig. S1). As seen previously, we found that under continuous dark conditions, Col tip angles narrowed as branches bent upward over time (significant average angle change of − 53.3, Fig. 1A and B, and [10]), while tip angles remained wider and more horizontal under continuous light (Fig. 1A and B). Branch angles, however, changed very little in either condition, regardless of genotype (Fig. 1C). These findings were visualized using time-lapse videos, made from images taken every 10 min for 72 h in each light condition (Additional file 2 and Additional file 3).

At the start of the experiment (time 0), *lazy1* mutant branch angles were wider, as reported previously [19], and tip angles began slightly downward (Fig. 1). In response to dark, tip angles narrowed (Fig. 1A and B). While this behavior was similar to Col, the change in angle was much greater (significant average angle change of − 153.1, Fig. 1B). *lazy1* mutants displayed an opposite response under continuous light, growing downward and exhibiting a widening tip angle with continued growth of the branch (significant average angle change of 65.0, Fig. 1A and B). This can further be seen in time-lapse videos in each light condition (Additional file 4 and Additional file 5). We previously hypothesized that *TAC1* may be a negative regulator of *LAZY1* [17]. Therefore, to address whether having a functional *TAC1* gene in *lazy1* mutant plants was necessary for this response to light, we assessed phenotypes of *tac1 lazy1* mutants under these conditions. *tac1 lazy1* mutants phenocopied *lazy1* mutants after 72 h in both light conditions (Additional file 1: Fig. S2), suggesting that *TAC1* is not required for the *lazy1* response to light.

Triple and quadruple *lazy/dro* mutants showed slightly wider initial branch angles than *lazy1* single mutants, but similar initial tip angles (Fig. 1). In constant dark conditions, triple and quadruple mutants exhibited very little change in tip angle (Fig. 1A and B). In light, tip angles showed some widening on average, but the response was variable and no significant change from 0 to 72 h was found (Fig. 1B). This downward growth, regardless of light condition, can be seen with finer dynamics in time-lapse videos of triple *lazy/dro* mutants (Additional file 6 and Additional file 7). This suggests that the loss of multiple *LAZY/DRO* genes reduces or eliminates upward branch tip growth and bending responses to darkness.

To investigate the effect of light fluence on branch and tip angles, we transferred plants to low- and high-light conditions (40 and 180 μmol m^−2^ s^−1^, respectively) for 72 h, maintaining a long-day photoperiod. Responses to low light were similar to constant dark conditions, and high light similar to constant light conditions. Under low light, Col tip angles narrowed significantly (significant average angle change of − 23.0°, Additional file 1: Fig. S3), as did *lazy1* tip angles, but to a greater extent (significant average change of − 100.4°, Additional file 1: Fig. S3). Growth in high light resulted in a widening of Col tip angles (19.6° average change, Additional file 1: Fig. S3), and downward growth in *lazy1* (48.7° average change, Additional file 1: Fig. S3), again approximating phenotypes of higher-order *lazy/dro* mutants. The quadruple *lazy/dro* mutant showed no significant change in either light condition. Together with the constant light and dark responses, this suggests that tip angle changes are a response to the total amount of light, rather than the photoperiod, and that loss of multiple *LAZY/DRO* genes leads to a reduction or loss in this response.

Unlike *lazy1* and *tac1*, single mutants of *dro1*, *dro2*, and *dro3* were reported to have no observable shoot phenotypes [22, 23], and thus we did not explore the effect of light environments on these single mutants. A *lazy6* single mutant has not yet been characterized, in part due to the lack of an available T-DNA insertion line; however, it was recently shown that *lazy1 dro1 dro2 dro3 lazy6* quintuple mutants showed no significant difference in branch angle changes from quadruple mutants in a gravitropism time course [32]. In a pLAZY6::GUS reporter line, we observed strong *LAZY6* gene expression in the shoot vasculature through 4 weeks of growth (Additional file 1: Fig. S4) indicating a potential function in GSA. Therefore, we engineered a *lazy6* CRISPR mutant to observe its branch angle phenotype in response to light changes. As a single mutant, we saw no significant difference in shoot architecture from Col in light or dark conditions (Additional file 1: Fig. S2), while *lazy1 lazy6* mutants showed similar branch angles and tip angles to *lazy1* mutants (Additional file 1: Fig. S2). This suggests that loss of *LAZY6* does not significantly impact GSA in response to light.

To determine whether constitutive *LAZY/DRO* gene expression could rescue or alter light-induced changes in branch orientation of *lazy1* mutants, we overexpressed *LAZY1*, *DRO1*, or *LAZY6* in the *lazy1* background and compared phenotypes at 72 h in response to light conditions. It was reported that expressing *LAZY1* under its native promoter in a *lazy1* mutant background only partially rescued the GSA phenotype [19, 32]. We found this to be true when overexpressing *LAZY1* in a *lazy1* mutant background as well, both in light and dark conditions (Additional file 1: Fig. S5). We also observed an upward leaf curling phenotype, which has been described for other IGT genes [20, 30], suggesting that the overexpression construct is at least partially functional (Additional file 1: Fig. S5). Next, we overexpressed *DRO1* in a *lazy1* mutant background and found that it not only rescued the branch angle phenotype of *lazy1*, but also phenocopied both branch angle and leaf curling phenotypes conferred by *DRO1* overexpression seen in both Col and *dro1* mutant backgrounds (Additional file 1:Fig. S5 and [20]). These lines exhibited narrow, upward-growing branches similar to a *tac1* mutant [17]. Upon growth in continuous light, branches responded by growing at a wider angle. Growth in the dark, however, had little to no impact, likely because branch angles were already near vertical (Additional file 1: Fig. S4A and B). In contrast to *DRO1*, *LAZY6* overexpression only partially rescued *lazy1* branch angle phenotype, suggesting the protein has some limited functional capacity to influence GSA when ectopically expressed. Branches grown in continuous light responded by growing downward with wider tip angles, but not to the extreme of the *lazy1* mutant, and dark-grown plants exhibited a phenotype similar to Col. These lines did not show a pronounced leaf curling phenotype indicating potential functional differences between *LAZY6* and *DRO1* or *LAZY1* (Additional file 1: Fig. S5C).

**Fig. 2:**
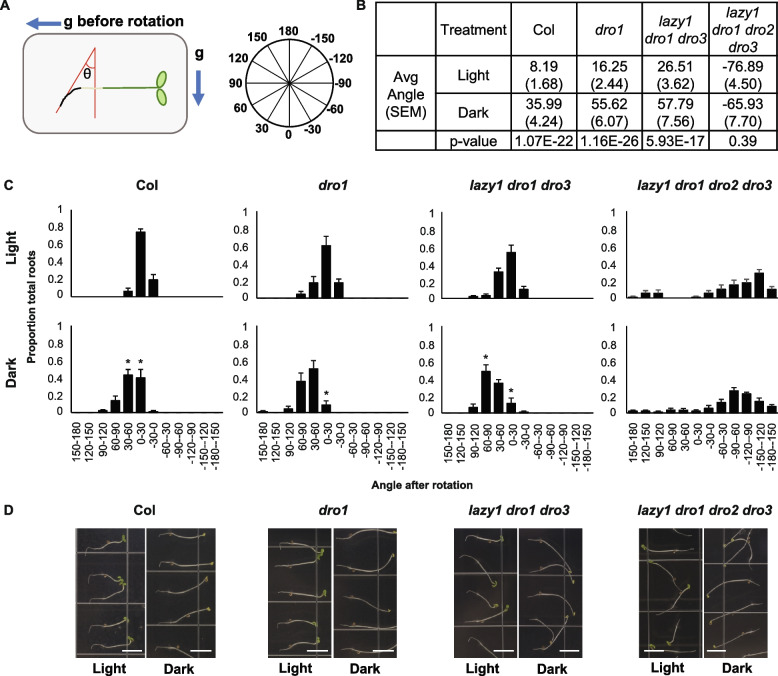
Loss of multiple *LAZY* and *DRO* genes confers root angle insensitivity to changes in light. **A** Schematic of experiment and measurements. Etiolated seedlings were rotated 90° and grown for 24 h in either constant light or dark and root tip angles were measured with respect to the new gravity vector. Root angles were measured and binned into intervals of 30° around a circle. g indicates the gravity vector. **B** Root tips of Col, *dro1*, and *lazy1 dro1 dro3* show a significant shift in average angle post-rotation when grown in light versus dark, while loss of four *LAZY* and *DRO* genes (*lazy1 dro1 dro2 dro3*) shows no significant difference. Student’s *t*-test was used to test for significance, using four replicates of 21–24 seedlings (one whole plate with four rows of 6 seedlings) each. **C** Histogram showing the proportion of total roots for each genotype corresponding to the angular position around a circle as shown in **A**. Asterisks indicate a significant difference (*p* < 0.05) of dark-grown seedlings as compared to light for each interval, using Student’s *t*-test with a Hommel *P*-value correction for multiple testing. Error bars indicate ± standard error of the mean (SEM) of the four replicates, each containing 21–24 seedlings. **D** Representative images of light- and dark-grown seedlings. White scale bars indicate 5-mm length

Recent work highlighted a difference in primary root growth angle between Col etiolated seedlings grown in 24 h of either continuous light or dark upon 90-degree rotation of plates [11]. To test whether progressive loss of *LAZY* and *DRO* genes influences this response as well, we grew Col, *dro1*, *lazy1 dro1 dro3*, and *lazy1 dro1 dro2 dro3* mutant seedlings in the dark for 3 days to allow etiolated growth, then transferred plates to either continuous light or dark conditions and rotated them 90° (Fig. 2A). After 24 h in these conditions, primary root angles were significantly different between the two conditions for Col. Each successive loss of IGT genes led to wider angles, demonstrated by progressively larger overall means and a shift in the distribution of *dro1* and *lazy1 dro1 dro3*; however, there was still a significant difference between light and dark growth, similar to Col (Fig. 2B and C). In contrast, quadruple *lazy1 dro1 dro2 dro3* mutants displayed a reverse growth direction and showed no difference in either mean root angle or distribution of angle intervals (Fig. 2B and C, suggesting that *LAZY* and *DRO* genes are collectively required for root angle reorientation responses to changes in light regimes.

### Influence of dark and light conditions on LAZY and DRO gene expression dynamics

To address the potential influence of light regimes on LAZY and DRO gene expression, we first used the cis-element database, AGRIS AtCisDB, to identify light-, circadian- and auxin-related cis-elements in the promoters of *LAZY* and *DRO* genes (Additional file 1: Fig. S6). All IGT genes contained light-related elements. GATA-box-containing cis-elements, which have been shown to be associated with light and circadian responsiveness [33, 34], appeared in all examined promoters. *LAZY1*, *DRO2*, *DRO3*, and *LAZY6* contained T-box elements, which are known to positively modulate light activation of a nuclear gene encoding a chloroplast protein [35]. *DRO1* and *DRO2* promoters contained an Auxin Response Element, known for ARF binding [36], and *DRO2* and *LAZY6* contained AtMyc2 sites, which have been implicated in blue light signaling and interaction with GATA-containing promoters [37, 38]. Cis-elements associated with phyA signaling (SORLREP and SORLIP) appeared in *DRO1*, *DRO3*, and *LAZY6*, and *LAZY6* additionally contained two G-box elements and a TGA1 site, both of which bind bZip transcriptions factors involved in light signaling [34, 39].

We next designed a set of experiments to measure changes in IGT gene expression in response to different light regimes and signaling pathways. We were able to design reliable and efficient quantitative PCR primers for four of the *LAZY* and *DRO* clade genes: *LAZY1* (At5g14090), *LAZY6* (At3g27025), *DRO1* (At1g72490), and *DRO2* (At1g19115) (Additional file 8: Table S1) [20, 22, 23, 40]. Unfortunately, after testing multiple primer pairs, we were unable to find efficient quantitative PCR primers that could consistently detect *DRO3* (At1g17400).

**Fig. 3:**
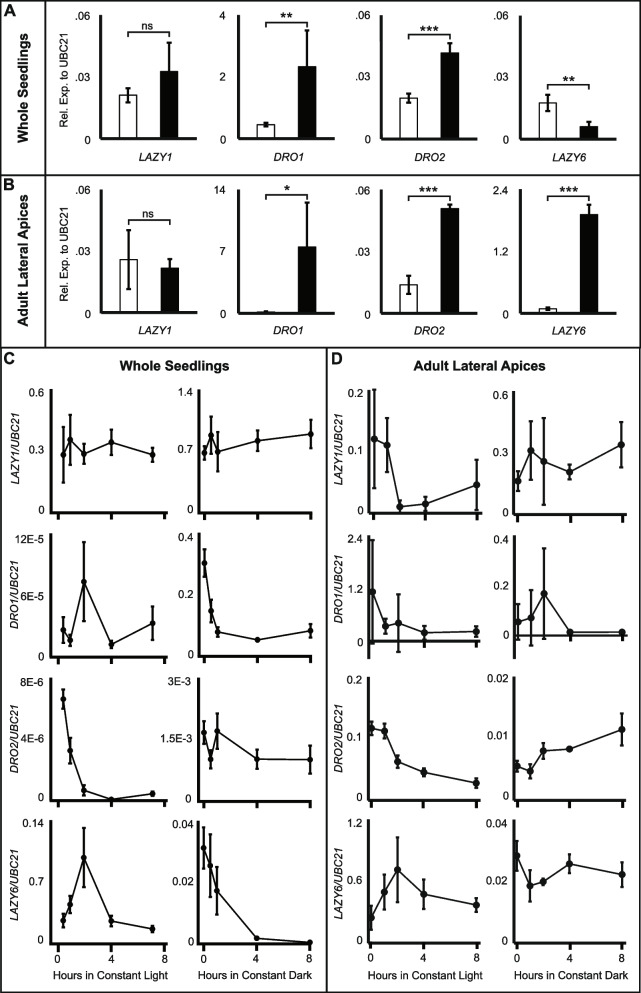
*LAZY* and *DRO* genes in seedlings and adult plants exhibit differential gene expression in response to continuous light and dark. **A** qPCR expression data for *LAZY1*, *DRO1*, *DRO2*, and *LAZY6* in whole Col seedlings grown for 10 days in 16L:8D cycles and moved to continuous light or dark for 72 h. **B** Expression for lateral apices (terminal 1–1.5 cm of branch tips) of adult plants moved to continuous light or dark for 72 h. Expression relative to housekeeping gene *UBC21* is reported. Error bars represent SD between four biological replicates of 10–12 pooled seedlings (one whole plate with 2 rows of 6 seedlings) per time point (seedlings) or four biological replicates of 2–5 pooled lateral apices (adults—pooled lateral apices for each individual plant). Student’s *t*-test was used to determine the significant difference between light and dark conditions at 72 h and is reported. *p < 0.05, **p < 0.01, ***p < 0.001. **C**, **D** Expression time-course of the first 8 h after plants are moved from 3-day dark growth to continuous light (left) or from 16L:8D cycles to continuous dark (right), in 10 dpg seedlings (**C**) or adult plants (**D**)

We first assessed gene expression at the end of 72 h of growth in constant light or dark, in both whole seedlings and lateral apices of adult Col plants. In seedlings, *LAZY1* expression was not significantly different between light conditions at 72 h of treatment, *DRO1* and *DRO2* both showed higher expression in dark, and *LAZY6* showed lower expression in the dark (Fig. 3A). Interestingly, when seedlings were separated into shoot and root tissues, similar results were seen in shoot tissues as whole seedlings, but the expression in root tissues either showed the opposite trend or no difference between continuous light and dark conditions (Additional file 1: Fig. S7). In lateral apices of adult plants, *LAZY1*, *DRO1*, and *DRO2* showed the same trend in expression as in seedlings, while *LAZY6* was higher in dark conditions at 72 h (Fig. 3B). To understand dynamic responses, we performed 8-h time course experiments to look at gene expression in the earlier time points after transfer to dark or light. Seedlings grown in 16L:8D (long day) conditions were transferred to continuous dark and sampled at 0, 0.5, 1, 2, 4, and 8 h after the transfer (Fig. 3C). A second set of seedlings, were grown in long-day conditions, transferred to continuous dark for 72 h, then returned to continuous light and sampled at similar intervals (Fig. 3C). After transfer to dark or light, *LAZY1* seedling expression showed little change over the time course experiment. *DRO1* expression initially decreased in the dark and spiked at 2 h in light conditions. *DRO2* showed no significant change in the dark but decreased over the first 2 h in the light. *LAZY6* decreased over the first 4 h of dark and spiked at 2 h in light (Fig. 3C). In apices collected from lateral branches of adult plants, *LAZY1* and *DRO1* expression was highly variable (Fig. 3D). A dip in *LAZY1* expression was measured after 2 h in light, as the pattern in darkness was similar to that seen in seedlings. *DRO1* expression was so variable across replicates that no change over the time period was observed in either condition. *DRO2* expression in adults overall showed similar changes in response to light as in seedlings, and *LAZY6* had similar responses to light, but little change was measured in darkness (Fig. 3D). These data demonstrate that *LAZY* and *DRO* genes are light responsive, showing differential dynamics in response to continuous dark and light treatments, and often show similar gene expression responses across life stages.

### Circadian influence on LAZY and DRO gene expression dynamics

**Fig. 4:**
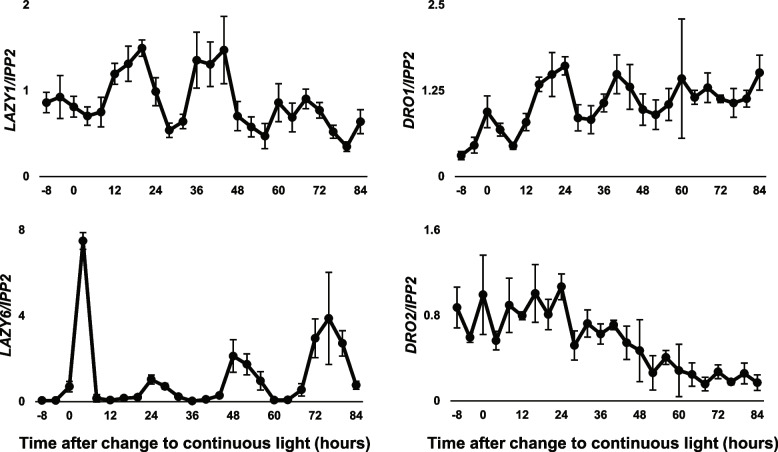
Gene expression of some IGT genes shows circadian rhythms. Time course of *LAZY1*, *LAZY6*, *DRO1*, and *DRO2* gene expression from seedlings entrained to a 12L:12D light cycle, then moved to continuous light for 84 h, relative to *IPP2* expression [41]. *LAZY1*, *LAZY6*, and *DRO1* show signatures of circadian rhythms, while *DRO2* steadily tapers with longer light exposure. Error bars represent SD between 4 biological replicates of 10–12 pooled seedlings (one whole plate with 2 rows of 6 seedlings) per time point

Several of the gene expression profiles we observed under continuous light or dark led us to investigate a potential role for circadian regulation in IGT gene expression. We decided to focus on seedlings, considering the number of samples needed for fine time resolution, and the higher relative level of variability in the adult data. To test this, seedlings were entrained to 12L:12D cycles, then transferred to continuous light conditions and sampled every 4 h for 3.5 days (Fig. 4), following established protocols for circadian experiments [41]. *LAZY1*, *LAZY6*, and *DRO1* expression exhibited peaks with near-24-h periods, shifting slightly over time (Fig. 4). In contrast, *DRO2* showed a weaker pattern and instead appeared to steadily drop throughout the duration of the continuous light treatment.

### LAZY and DRO gene expression responds differentially to light waveband and photoreceptors

**Fig. 5:**
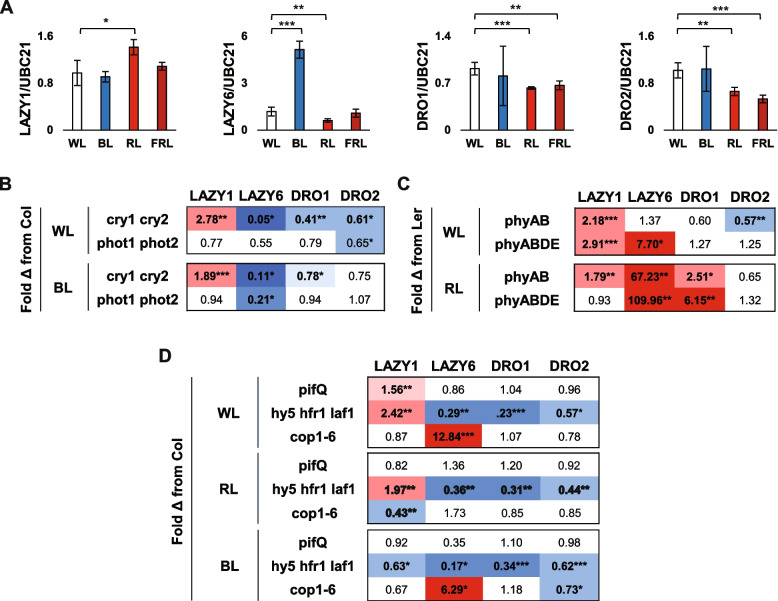
Effect of light color and photoreceptor-mediated signaling mutants on *LAZY* and *DRO* gene expression. **A** qPCR expression data for Col seedlings grown in 16L:8D cycles and moved to continuous W, R, B, or FR light for 72 h. Error bars represent SD between 4 biological replicates of 10–12 pooled seedlings (one whole plate with 2 rows of 6 seedlings) per time point. **B** Fold change expression differences between Columbia wild-type and blue light signaling mutants, *cry1 cry2* and *phot1 phot2*, moved from 16L:8D light cycles to either continuous WL or BL. Significant changes from Col in each light color condition are reported. **C** Fold change expression differences between Columbia wild-type and red light signaling mutants, *phyAB* or *phyABDE*, moved from 16L:8D light cycles to either continuous WL or RL. Significant changes from Ler in each light color condition are reported. **D** Fold change expression differences between Columbia wild-type and light signal integration mutants, *pifQ*, *hy5 hfr1 laf1*, and *cop1-6*, moved from 16L:8D light cycles to either continuous W, R, or B light. Significant changes from Col in each light color condition are reported. Expression values were calculated relative to *UBC21* and represent the average between 4 biological replicates of 10–12 pooled seedlings (one whole plate with 2 rows of 6 seedlings) per comparison. Significant changes are shaded blue for downregulation and red for upregulation. *P < 0.05, **P < 0.01, ***P < 0.001, using Student’s *t*-test, comparing to the control in each light treatment

To assess the effect of different light wavebands and photoreceptor-mediated pathways on the expression of *LAZY* and *DRO* genes, we began by transferring 10-day-old seedlings to continuous Blue (B), Red (R), or Far-Red (FR) light for 3 days (Fig. 5A). A change from 16L:8D White (W) light to continuous B light only had a significant effect on *LAZY6* expression, which was upregulated more than threefold. R light had a significant effect on all genes, leading to an increase in *LAZY1* expression, but a decrease in the other three genes. FR light only had a significant effect on the *DRO* genes, leading to a significant decrease in *DRO1* and *DRO2* expression (Fig. 5A). Together, this demonstrated a differential response of *LAZY* and *DRO* gene expression to different wavelengths of light.

To parse the potential regulatory influence of known photoreceptor-mediated pathways, we looked at expression in signaling mutant backgrounds associated with cryptochrome (cry), phototropin (phot), and phytochrome (phy) pathways, as well as downstream signaling genes known to integrate various light signals. Mutants were transferred from 16L:8D conditions to continuous W, B, or R light for 3 days, and expression fold change compared to controls in each light condition was calculated. While *LAZY1* showed no major change in expression in response to B light alone, its expression significantly increased in a *cry1 cry2* background, both in W and B light treatments (Fig. 5B). In contrast, *LAZY6* exhibited upregulation in B light treatments and downregulation in *cry1 cry2* mutants under both W and B light. *DRO1* and *DRO2* expression decreased in a *cry1 cry2* background under W light, but only *DRO1* decreased slightly under B light. The loss of *phot1* and *phot2* had little significant effect on expression for most genes and light conditions, with the exception of *LAZY6* downregulation in B light and *DRO2* downregulation in W light (Fig. 5B).

Loss of two (*phyA* and *phyB*) or four *phys* (*phyA*, *B*, *D*, and *E*) had a largely positive effect on *LAZY* and *DRO* genes (Fig. 5C). *LAZY6* showed the most dramatic increases, between 7.7- and 110-fold in response to the loss of the *phy* genes, compared to controls under the same conditions. *LAZY1* increased significantly in *phy* mutant backgrounds in the W light treatment, but to a lesser extent under R light conditions. *DRO1*, in contrast, showed no response to the absence of functional *phy* genes in W light conditions, but dramatic increases under R light. *DRO2* exhibited minor changes in expression, only significantly downregulated in the *phyA phyB* background in W light.

A number of genes are known to act downstream of multiple light signaling pathways, acting as a signal integration center [42–44]. These include *PHYTOCHROME INTERACTING FACTORS* (*PIFs*), *ELONGATED HYPOCOTYL 5* (*HY5*), *LONG HYPOCOTYL IN FAR RED 1* (*HFR1*), *LONG AFTER FAR-RED LIGHT 1* (*LAF1*), and *CONSTITUTIVE MORPHOGENESIS 1* (*COP1*). Similar to the photoreceptor mutants, mutations in these genes showed differential effects on *LAZY* and *DRO* gene expression (Fig. 5D and E). Loss of four of the *PIF* genes (*PIF1*,*3*,*4*,*5*) in the *pifQ* mutant had little significant effect on the expression of IGT genes under all light conditions, with the exception of *LAZY1* upregulation in W light. *LAZY1* also showed upregulation in the triple *hy5 hfr1 laf1* mutant background under W and R light and downregulation in B light, while *LAZY6*, *DRO1*, and *DRO2* were downregulated in this background in all light conditions. The *cop1-6* mutant background resulted in the downregulation of *LAZY1* in red light and *DRO2* in B light, and a large, significant upregulation of *LAZY6* in W and B light.

## Discussion

IGT genes play important roles in setting the gravitropic set-point angle (GSA), as well as the capacity to respond to changes in orientation (gravitropism). Most studies on IGT genes to date have focused on their roles in GSA and gravitropism response without varying light conditions. Our phenotypic analyses of adult plants highlight the roles of IGT genes for setting branch growth/bending angles in response to light, independent of changes in gravity, while analysis of root tips demonstrates their necessity for proper response to simultaneous changes in light and gravity. We found that *lazy1* has hyper-sensitive responses to continuous light and dark, exhibiting a greater tip angle response to darkness than Col, and a greater tip angle response to continuous light than the triple and quadruple IGT gene mutants (Fig. 1). Further, loss of three (*LAZY1*, *DRO1*, and *DRO3*) or four (*LAZY1*, *DRO1*, *DRO2*, and *DRO3*) IGT genes led to a loss of response, in both shoot and roots, to changes in light conditions (Figs. 1 and 2). We found that ectopic expression of *DRO1* or *LAZY6* can rescue or partially rescue the light-response phenotypes of a *lazy1* mutant, but that neither *tac1* nor *lazy6* enhanced them in our conditions. It has been previously well-established that light plays a role in setting GSA, and the work presented here suggests that IGT genes play a crucial role in determining gravity response in a light-dependent fashion [1, 9–11].

Time-lapse videos showed the dynamics and context of how branches are moving and changing growth angles over time. They also revealed the differential duration of circumnutation movements between genotypes (Additional files 2, 3, 4, 5, 6, and 7). In rice, *lazy1* coleoptiles exhibit highly impaired circumnutation [45]. Previous work in Arabidopsis demonstrated that Col and *lazy1* showed similar circumnutation activity when monitored from above for 20 h [19]. While we were unable to measure movement from above, time-lapse videos revealed that Col shoots circumnutate longer than *lazy1* shoots in a light-dependent fashion. Col shoot tips show very active movement in constant darkness, but slow to a stop around 44–48 h. In constant light, the main stems continued moving throughout the time course, with only a few pauses (Additional files 2 and 3). By comparison, *lazy1* shoot tips in constant darkness stop moving after ~ 24 h. In light, all *lazy1* shoots started growing towards gravity relatively quickly, making it difficult to ascertain movement patterns; however, the main stems appeared to keep moving for ~ 12 h. Triple *lazy/dro* mutants moved very little in the dark. In light, the main stems circumnutated for the first few hours, but like *lazy1*, started growing downward towards gravity very quickly. As *LAZY1* and *DRO1* expression showed measurable circadian signatures, it warrants future experimentation to determine the role these genes play in the regulation of circumnutation. The circadian expression profiles of these genes further raise questions about gravity sensitivity throughout the day.

It is noteworthy that in our experiments, branch crotch angles changed very little, regardless of condition, while tip angles were the responsive variable. Responsiveness of branch angle to environmental factors is likely due to multiple factors, including age/length of the lateral branch, growth conditions leading up to the environmental stimulus being tested, and duration of growth under the changed environmental conditions. For our experiments, we used plants that had reached ~ 15–22 cm in height (usually ~ 6–7 weeks of growth in long-day, 20 °C conditions), and only analyzed branches that measured ~ 3–12 cm at the start of the experiment. This may have resulted in plants that had relatively rigid branch angles and the more pliable branch tip tissue was more competent to respond. Further, our experiments typically lasted 72 h in the altered light conditions. Other work has found that plant growth in low-light and high-temperature settings resulted in differences in branch angle [9]. Based on the methods used, these plants were grown for 14 days under these conditions and were likely transferred at a young age [9]. It is also noteworthy that under these conditions for a 14-day period, plants exhibited thinner, weaker inflorescence stems and branches. Experiments using a clinostat to assess gravity responses found that shorter, younger branches (0–2 cm in length) displayed greater curvature, and thus a greater response, than longer branches (2–5 cm or > 5 cm) [46]. It is possible that with younger branches and longer experimental time, we may have seen significant changes in branch angles, in addition to tip angles.

The influences of light and gravity on plant growth are notoriously difficult to tease apart, and what role the IGT genes play, if any, in the relation between gravitropism and phototropism is unclear. Previous work with higher-order *lazy/dro* mutants has described phototropic responses to unilateral blue light [22, 23]. In both etiolated seedlings and adult inflorescence stems, triple or quadruple mutants (*lazy1 dro1 dro3* or *lazy1 dro1 dro2 dro3*) showed exaggerated responses to blue light, bending towards the blue light to a greater degree than wild-type plants [22, 23]. Phototropic bending in hypocotyls is thought to be due to a redistribution of PIN3, downstream of activation of phot1 and dephosphorylation of NPH3, resulting in an asymmetric auxin concentration across the hypocotyl [47]. In *lazy/dro* triple mutant and *dro1* single mutant roots, PIN3 has been shown to localize more to the upper side of root tips after gravistimulation, in contrast to wild-type localization toward the lower side [22, 48]. Upon phototropic bending, hypocotyls and inflorescence stems are also bending in the direction of gravity. PIN3 redistribution to the upper side of these organs may occur in *lazy/dro* triple mutants, as seen in root tips, and may compound with the PIN3 redistribution already happening in response to blue light, offering a possible explanation for the exaggerated responses. As our experiments used overhead light, we were unable to look at the responses in lateral shoots to different light regimes using unilateral light. However, our data does demonstrate that *lazy/dro* triple mutant lateral branch tips grew towards gravity regardless of change in light level or photoperiod (constant light or dark), suggesting that they have lost the ability to respond to these stimuli.

Recent work has highlighted connections between light, plastids, statocytes, and the actin cytoskeleton [49]. Statocyte-specific expression of *ALTERED RESPONSE TO GRAVITY (ARG1)* and *ARG1-LIKE 2 (ARL2)*, genes localized to peripheral membranes and vesicle trafficking components, is required for gravitropic responses in the early phases of signal transduction in Arabidopsis [50]. ARG1 may also interact with the actin cytoskeleton [51]. An enhancer screen of *arg1* mutants revealed components of the Translocon at the Outer envelope membrane of Chloroplasts (TOC) complex, which may be involved in protein transport, to enhance the loss of gravity response, but these proteins are not localized to the same parts of the cell and further experiments suggest an indirect interaction between them [50, 52]. Both Arabidopsis DRO1 and DRO3 were shown to interact with a component of the TOC complex in a protein–protein interaction screen using a co-immunoprecipitation and LC–MS/MS approach and raised the hypothesis that IGT proteins could be candidates for TOC cargo [26]. The same protein-interaction screen further revealed several photosystem subunit and light-harvesting complex proteins, highlighting more interesting potential connections between IGT genes and light signaling [26].

RICE MORPHOLOGY DERTERMINANT (RMD), a rice actin-binding protein, further highlights links between light and gravity, involving the actin cytoskeleton and amyloplast sedimentation [3]. *rmd* mutants exhibited altered GSA phenotypes, reorientation responses, and amyloplast sedimentation in the light due to a disturbed actin cytoskeleton, but normal responses in the dark. Researchers found that *RMD* was diurnally expressed and bound to and negatively regulated by PIL15 and 16 in the dark, leading to a model where *RMD* is responsible for actin cytoskeleton reorganization in the light when *PIL* expression is reduced [3]. Amyloplast sedimentation after reorientation has been shown to be normal in *lazy1 dro1 dro3* triple mutants and thus is likely downstream of this step in gravity perception [22]. However, this work highlights additional IGT-independent gravity response pathways, which have been suggested previously [31].

Comparison of gene expression after multi-day growth in continuous light and dark revealed that, in adult lateral apices, expression of IGT genes other than *LAZY1* was significantly lower in light-grown plants (Fig. 3B). This relatively lower expression in constant light and higher expression in dark may explain the dramatic changes in *lazy1* mutant phenotypes in these conditions (Fig. 1). *lazy1* grown in continuous light phenocopied triple and quadruple mutants, with branch tips growing downward, which could be in part due to decreased expression of *DRO1*, *DRO2*, and *LAZY6*. Similarly, *lazy1* branch tips grown in continuous dark bent upwards, possibly due to increased expression of the other IGT genes. This is consistent with the ectopic expression of *DRO1* in a *lazy1* background that resulted in extremely narrow branch and tip angles while the ectopic expression of *LAZY1* or *LAZY6* only partially rescued the mutant phenotype (Additional file 1: Fig. S5 and [20]). The most likely explanation for these results is that the LAZY1/LAZY6 proteins are under distinct post-transcriptional regulation from DRO1 in the shoot or alternatively that the DRO1 protein has a higher level of gravity signaling activity. Regardless, the findings are consistent with mutant GSA phenotypes being mediated by light-induced changes in IGT expression.

Loss of photoreceptors and associated signaling pathway components influenced IGT gene expression in complex ways. Among B light photoreceptor mutants, *cry1 cry2* affected the expression of all IGT genes (Fig. 4B), with increases in *LAZY1* and decreases in *LAZY6*, *DRO1*, and *DRO2*, despite few genes responding to B light treatments (Fig. 4A). While this may suggest regulation by the cryptochrome pathway, this may alternatively reflect a change in the circadian period, which both *cry* and *phy* mutants are known to alter [53]. IGT gene expression showed similar responses to *hy5 hfr laf1* mutants (Fig. 4D). HY5 has been recently shown to mediate B light influence on the circadian clock, with mutants exhibiting shorter circadian periods [54]. HY5 has additionally been shown to bind to the *DRO1* promoter to alter expression in response to light, shaping root gravitropism [11]. Thus, responses to mutations in *CRY* and *HY5* genes may involve a combination of indirect B light or circadian influence, and direct HY5 regulation. While we have yet to clearly identify a *lazy6* mutant phenotype, *LAZY6* expression showed dynamic and dramatic responses to changes in light, light signaling mutants, and circadian rhythms. The *LAZY6* promoter contained several different cis-elements related to phyA signaling and bZip transcription factor binding, which is in line with its strong light-induced changes. Together with the finding that *LAZY6* overexpression partially rescues a *lazy1* mutant, these lines of evidence may point to a yet undiscovered light-related function. It should be noted that *DRO1* expression showed no change in a *pifQ* background, contrary to evidence shown previously; however, this may be explained by the difference in tissues analyzed and the light regimes used [11].

When separated into shoot and root tissues, seedlings exhibited differential expression patterns of all four IGT genes tested (Additional file 1: Fig S7). In seedling roots, *LAZY1* showed a large and significant decrease in dark compared to light, while both *DRO* genes showed no change, in contrast to increased expression in dark in shoot tissues. However, we know that *DRO* genes are more highly expressed and have the strongest mutant phenotypes in the root [20, 40] and that loss of *LAZY* and *DRO* genes decreased the difference between dark and light root tip growth angles (Fig. 2). The lack of *DRO* expression differences in light and dark-grown roots might suggest that light affects their protein activity and function, rather than gene expression. Indeed, much of the current work elucidating the mechanistic role of *DRO1* in setting root GSA has demonstrated that *DRO1* localization and protein interactions are central to this role. In shoot tissues, both in seedlings and adult lateral apices, *DRO1* and *DRO2* gene expression is higher in darkness than in constant light. This may reflect their role in light-dependent changes in tip angles in both Col and *lazy1* mutants. *LAZY1* expression also increases in darkness in seedling shoots, but not in adult tissues. This may again reflect the influence of light on protein activity rather than expression. For all IGT genes, light may influence both expression and protein function in different tissues and processes.

While a combination of intact *LAZY1*, *DRO1*, and *DRO3* are required for sensitivity to changes in light regimes, the effect of *DRO2*, *LAZY5*, and *LAZY6* are less clear. Triple *lazy1 dro1 dro3* mutants exhibit an extreme reverse gravitropic growth defect and show insensitivity to constant light, constant dark, high light, and low light, despite containing intact *LAZY5*, *LAZY6*, and *DRO2* genes. This raises the question of *LAZY* and *DRO* individual genetic contributions to the light sensitivity of GSA. While not exposed to changes in day length, recent experiments show that higher order mutant seedlings containing *dro2*—both *lazy1 dro1 dro2 dro3* and *dro1 dro2 dro3* (*atlazy1,2,3,4* and *atlazy2,3,4* from [23])—have more severe hypocotyl and root gravitropic defect than counterparts lacking the *dro2* mutation, suggesting an essential role in root gravitropism for *DRO2*. In shoots of adult plants, a *lazy1 dro2* mutant appears to have a similar phenotype to *lazy1* alone, and *dro1 dro2* and *dro2 dro3* mutants show little if any shoot phenotype [23]. However, we were not able to test these mutant’s responses to changes in light, and further study is required to understand how *DRO2* contributes to the light response phenotype. Due to a lack of availability of a *LAZY6* T-DNA insertion mutant, a higher order mutant has only recently been published and demonstrated that loss of *LAZY6* did not enhance the gravitropic defect of loss of *LAZY1*, *DRO1*, *DRO2*, and *DRO3* together in adult shoots (*atlazy1,2,3,4,6* in [32]). We found that *lazy6* did not significantly enhance the *lazy1* light response phenotype, but *LAZY6* overexpression could partially rescue (Additional file 1: Fig. S5). Recent experiments demonstrate that seedling primary root gravitropism is light sensitive [11], although it is currently unclear how individual IGT genes contribute to this phenotype. Future light experiments with different combinations of higher-order mutants will help to clarify the individual roles of these genes in modulating gravity in response to light signals.

This work supports a model in which plants have evolved complex signal integration of light and gravity, where light influences gravity responses in part through changes in the expression of IGT genes. Here and in previous studies, daylength, light wavelength, photosynthesis, and the circadian clock all play roles in influencing the expression of gravity response regulators, including the IGT genes [3, 10, 11, 26, 29, 50]. This may partially explain the high degree of plasticity and ability of plants to alter their growth orientation by taking into account and prioritizing multiple light-related inputs. It also highlights the need to monitor gravity response when studying phototropism and vice versa, since the two may be strongly interconnected.

## Conclusions

Together, our findings support a model in which *LAZY* and *DRO* genes are collectively required for light-responsive changes in the capacity to respond to gravity. The complex expression patterns of IGT family genes suggest that light signals contribute to root and branch orientation in part through differential regulation of IGT gene family members. Future work will be necessary to uncover the precise roles of individual IGT gene family members in light response and how this integrates with responses to other environmental stimuli.

## Methods

### Plant materials and growth conditions

The Columbia (Col-0) ecotype was used as wild-type in all experiments. The *lazy1* mutant used here was a T-DNA insertion line (GABI_591A12) obtained from NASC (https://arabidopsis.info). The *tac1 lazy1* double mutant was generated by crossing the *tac1* and *lazy1* single SALK mutants (*tac1*, T-DNA insertion line CS825872 from ABRC) as described in [30]. *lazy6* and *lazy1 lazy6* lines were generated by transforming LAZY6 CRISPR constructs into Col-0 and *lazy1* backgrounds, respectively. Briefly, for CRISPR constructs, the *LAZY6* target sequence was identified using crispr-plant (http://www.genome.arizona.edu/crispr/) and cloned into the pHEE401E vector [55] using Gibson cloning [56]. The resulting *LAZY6* CRISPR line shown here contains a single G insertion at nucleotide position 229 of the cDNA, resulting in a premature stop after the 80th codon, and was phenotypically representative of multiple CRISPR mutants. The 35S::DRO1 and 35S::LAZY1 constructs were cloned as preciously described [20, 30]. Similarly to these, the 35S::LAZY6 construct was cloned by ligating the LAZY6 (At3g27025) CDS into the multiple cloning site downstream of the 35S promoter of a modified pBIN-AFRS expression vector [57]. All three constructs were transformed into *lazy1* mutant Arabidopsis plants via floral dip [58]. A list of all primers used for cloning and genotyping can be found in Additional file 8: Table S2. Six to 10 T2 lines were evaluated for phenotypes and expression, and 2–3 representative T3 lines were chosen for further analysis. For quantification of plants containing *DRO1* and *LAZY6* overexpression constructs, one representative line was used, as all lines demonstrated similar phenotypes. Signaling mutants *phyA phyB* and *phyA phyB phyD phyE* [59], *cry1 cry2* [60], *phot1 phot2* [61], *cop1-6* [62], *pif1 pif3 pif4 pif5* [63], and *hy5 hfr1 laf1* [64] were previously described. *lazy1 dro1 dro3* (*lazy1 lazy2 lazy4*) and *lazy1 dro1 dro2 dro3* (*lazy1 lazy2 lazy3 lazy4*) were generated and described previously [23]. For seedling expression studies, seeds were surface sterilized and sown on square plates containing half-strength Murashige and Skoog (MS) medium and 0.8% bactoagar, and grown vertically, following our standard lab practice. Once sown, seedlings were stratified at 4 °C in the dark for 2 days, then placed in growth chambers at 20 °C with a 16-h light/8-h dark photoperiod (~ 100 μmol m^−2^ s^−1^). For adult phenotyping studies, 14-day-old seedlings were transplanted to soil and allowed to grow in these conditions until bolting. All plant material was used in accordance with all local, national, and international guidelines, legislations, and permissions.

### Branch angle phenotypes

For shoot branch phenotypes, seedlings were grown for 2 weeks on plates, then transplanted into 4-in. pots containing Metromix 360 or Sunshine Mix 4 soil supplemented with vermiculite (Sun-Gro Horticulture, http://www.sungro.com) and grown until bolts had lateral branches at least 3 cm in length and inflorescence stems reached ~ 15–22 cm in height. Plants were then transferred to continuous light or dark conditions for 72 h. Images were taken using a Canon EOS Rebel T3 camera (http://global.canon/en/index.html). For time course experiments in the dark, images were taken in the dark and lit with green light. For analysis of all time course experiments, individual pots were rotated for each image of an individual branch, such that the branch was perpendicular to the camera, allowing for accurate angle analysis from the image. For endpoint analyses in Additional file 1: Fig. S2 and S5, plants were preserved by taping down flat to paper, to aid in accurate angle analysis. For statistical analysis of phenotypes, Student’s *t*-tests were used and standard error of the mean is reported.

### Root angle phenotypes

For root angle phenotypes, seedlings were sown on 0.5 × MS plates and stratified at 4 °C for 2 days. Plates were removed from the cold, germinated in light for 2 h, and grown in the dark for 3 days to cause etiolation. At this point, plates were rotated 90° and either moved to continuous light or kept in continuous dark for 24 h. Plates were then imaged, and primary root gravitropic response angles were measured using Image J. Statistics were calculated using Excel and R, for Student’s *t*-tests and multiple comparison corrections, respectively, and standard error of the mean is reported. Roots growing in contact with agar will grow, rather than bend, in response to gravity changes; thus, we did not capture images of plates before rotation, as the angle of growth before rotation can be seen above the point of reorientation.

### RNA extraction and qPCR

For seedling studies, seedlings were grown on vertical plates for 10–14 days. Four biological replicates were used. Each biological replicate consisted of a plate of 10–12 seedlings. For adult lateral apices, plants were grown to 15–22 cm in height and ~ 1–1.5 cm of all lateral branch tips were collected and pooled for each plant. Each biological replicate consisted of an individual adult plant. RNA was extracted using a Directzol RNA Extraction Kit (Zymo Research, http://www.zymoresearch.com). qPCR was performed as previously described by [10]. Briefly, each reaction was run in triplicate using 50 ng of RNA in a 12 μl reaction volume, using the Superscript III Platinum SYBR Green qRT-PCR Kit (ThermoFisher Scientific, https://www.thermofisher. com). The reactions were performed using an ABI7900 qPCR machine (Applied Biosystems, now ThermoFisher Scientific, https://www.thermofisher.com) or a Bio-Rad CFX384 (Bio-Rad, https://www.bio-rad.com/). Quantification of Arabidopsis samples was performed using a standard curve derived from a serially diluted standard RNA, run in parallel on each plate. Analysis was performed using either the standard curve method (Figs. 4 and 5) or the Pfaffl method (Fig. 3 and Additional file 1: Fig. S6, [65]). For the standard curve method, the Applied Biosystems SDS 2.4 software (now ThermoFisher Scientific, https://www.thermofisher.com) used the serially dilute RNA to determine the slope and subsequent quantity of expression of samples on each plate. *UBC21* was used as an internal control to normalize expression in light experiments as it was identified as a highly constitutive gene [66], and *IPP2* was used for circadian experiments, as it has been used as a standard for circadian experiments previously [41]. When comparing expression between two conditions, Student’s *t*-tests were used and the standard deviation of four replicates is reported. A list of primers used in this study can be found in Additional file 8: Table S2.

### Light and time-course expression experiments

For seedling light experiments, plants were grown for 10 days on vertical plates in 16:8 h long daylight conditions in a growth chamber before transfer to experimental light conditions. For comparisons between light and dark, plates were moved to chambers with either continuous light or continuous dark conditions for 72 h, then whole seedlings were collected and flash frozen at 10.00 am [Zeitgeber time (ZT4)]. For comparisons between light wavebands, plates were moved to chambers with continuous white (W), red (R; 660 nm), blue (B; 480 nm), or far red-light (FR; 738 nm) for 72 h and whole seedlings were collected at 10.00 am (ZT4). Matching growth chambers fitted with W, R, B, and FR LED lamps from PARsource (http://parsource.com) were used for light waveband experiments. For photoreceptor mutant experiments, seedlings were used, as many adult phenotypes make the plants difficult phenotypes to maintain. For circadian experiments, seedlings were grown for 10 days in 12L:12D light cycles, then transferred to continuous light and collected every 4 h, as is standard for circadian studies, for 84 h. For the identification of light-related cis-elements in the IGT gene promoters, we used the AGRIS AtCis Database (https://agris-knowledgebase.org/AtcisDB/). 

### Supplementary Information

**Additional file 1:** **Fig. S1.** Cartoon description of angle measurements used in this study. **Fig. S2.** *tac1 lazy1* and *lazy1 lazy6* mutants largely phenocopy *lazy1* mutants in constant light and dark conditions. **Fig. S3.** Quadruple *lazy/dro* mutant tip angles exhibit insensitivity to high and low light, while *lazy1* mutants appear hypersensitive. **Fig. S4.** *LAZY6* is expressed in shoot vasculature through 28 dpg. **Fig. S5.** Overexpression of *LAZY1*, *DRO1*, and *LAZY6*, alters light-induced branch angle phenotypes in a *lazy1* mutant background. **Fig. S6.** *LAZY* and *DRO* genes contain several types of light-related cis-elements. **Fig. S7.** *LAZY* and *DRO* gene expression shows differing responses to light changes in seedling shoot and roots.

**Additional file 2.** Time lapse video of Col wild type plants grown in constant dark for 72 h.

**Additional file 3.** Time lapse video of Col wild type plants grown in constant light for 72 h.

**Additional file 4.** Time lapse video of *lazy1* mutant plants grown in constant dark for 72 h.

**Additional file 5.** Time lapse video of *lazy1* mutant plants grown in constant light for 72 h.

**Additional file 6.** Time lapse video of *lazy1 dro1 dro3* triple mutant plants grown in constant dark for 72 h.

**Additional file 7.** Time lapse video of *lazy1 dro1 dro3* mutant plants grown in constant light for 72 h.

**Additional file 8:** **Table S1.** Table of IGT/LAZY family gene names and Arabidopsis IDs from current and recent studies. **Table S2.** Primers used in this study.

**Additional file 9.** Raw data used for Figs. 1, 2, 3, 4, 5 and Additional file 1: Figs. S2, S3, S5, and S7.

## Data Availability

Biological material developed for this study (seeds, plasmids) may be requested from the authors. All data generated or analyzed during this study are included in this published article and its supplementary information files. Individual data for Figs. 1, 2, 3, 4, and 5 and Additional file 1: Fig S2, S3, S5, and S7 are provided in Additional file 9.

## Abbreviations

GSA: Gravitropic set-point angle
16L:8D: 16-Hour light, 8-h dark, also referred to as “long day”
T-DNA: Transfer DNA, in this case, lines in which sections of the Ti plasmid from agrobacterium have been inserted into specific genes, resulting in a mutation of that gene
GUS: β-Glucuronidase reporter gene
CRISPR: Clustered regularly interspaced short palindromic repeats
AGRIS AtCisDB: Arabidopsis Gene Regulatory Information Server, Arabidopsis cis-regulatory element database
PCR: Polymerase chain reaction
12L:12D: 12-Hour light, 12-h dark
W, B, R, and FR light: White, Blue, Red, and Far-Red light
LC–MS/MS: Liquid chromatography with tandem mass spectrometry
qPCR: Quantitative polymerase chain reaction
RNA: Ribonucleic acid
dpg: Days past germination
