# Supplementary material for: IGT/LAZY genes are differentially influenced by light and required for light-induced change to organ angle
Source: BMC Biol. 2024 Jan 17;22:8. doi: 10.1186/s12915-024-01813-4 (PMC10795295; doi:10.1186/s12915-024-01813-4)
Supplement: Supplementary file 1 — Additional file 1: Fig. S1. Cartoon description of angle measurements used in this study. Fig. S2. tac1 lazy1 and lazy1 lazy6 mutants largely phenocopy lazy1 mutants in constant light and dark conditions. Fig. S3. Quadruple lazy/dro mutant tip angles exhibit insensitivity to high and low light, while lazy1 mutants appear hypersensitive. Fig. S4. LAZY6 is expressed in shoot vasculature through 28 dpg. Fig. S5. Overexpression of LAZY1, DRO1, and LAZY6, alters light-induced branch angle phenotypes in a lazy1 mutant background. Fig. S6. LAZY and DRO genes contain several types of light-related cis-elements. Fig. S7. LAZY and DRO gene expression shows differing responses to light changes in seedling shoot and roots. [file 12915_2024_1813_MOESM1_ESM.pdf]

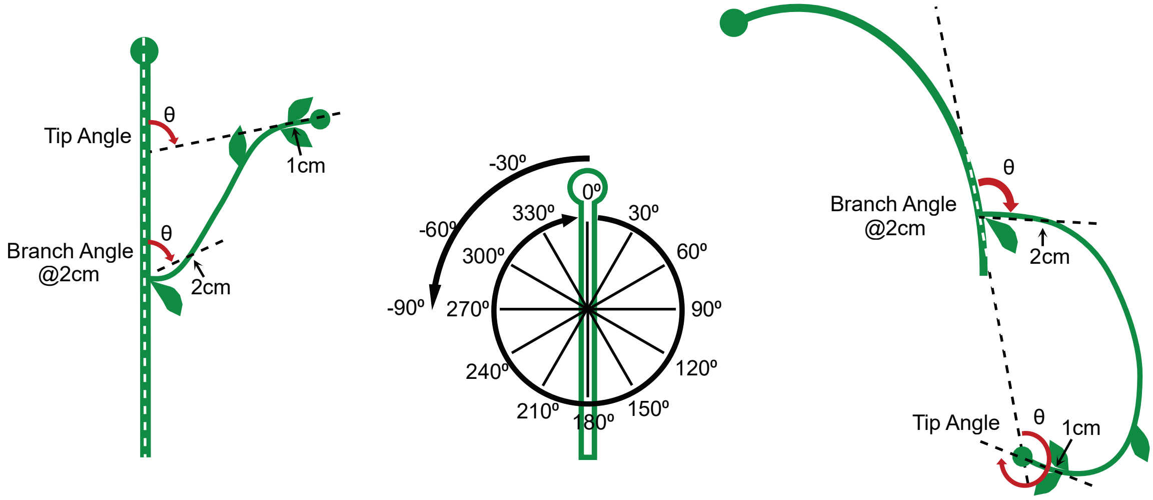

Figure S1. Cartoon description of angle measurements used in this study
To measure branch angles a line was drawn between the branch point and 2cm down the branch, and the angle of this line with respect to the angle of the stem above the branch point was recorded. To measure tip angles, a line was drawn tangent to the branch, 1 cm back from the branch tip, and the angle of this line with respect to the angle of the stem above the branch point was recorded. When branch continued to curl under as they grew and exceeded 180 degrees, we considered this a continuation of the angle (i.e. between 180-360), to differentiate between these and branches that had grown upward and narrowed into negative values (i.e. 0 to -180).

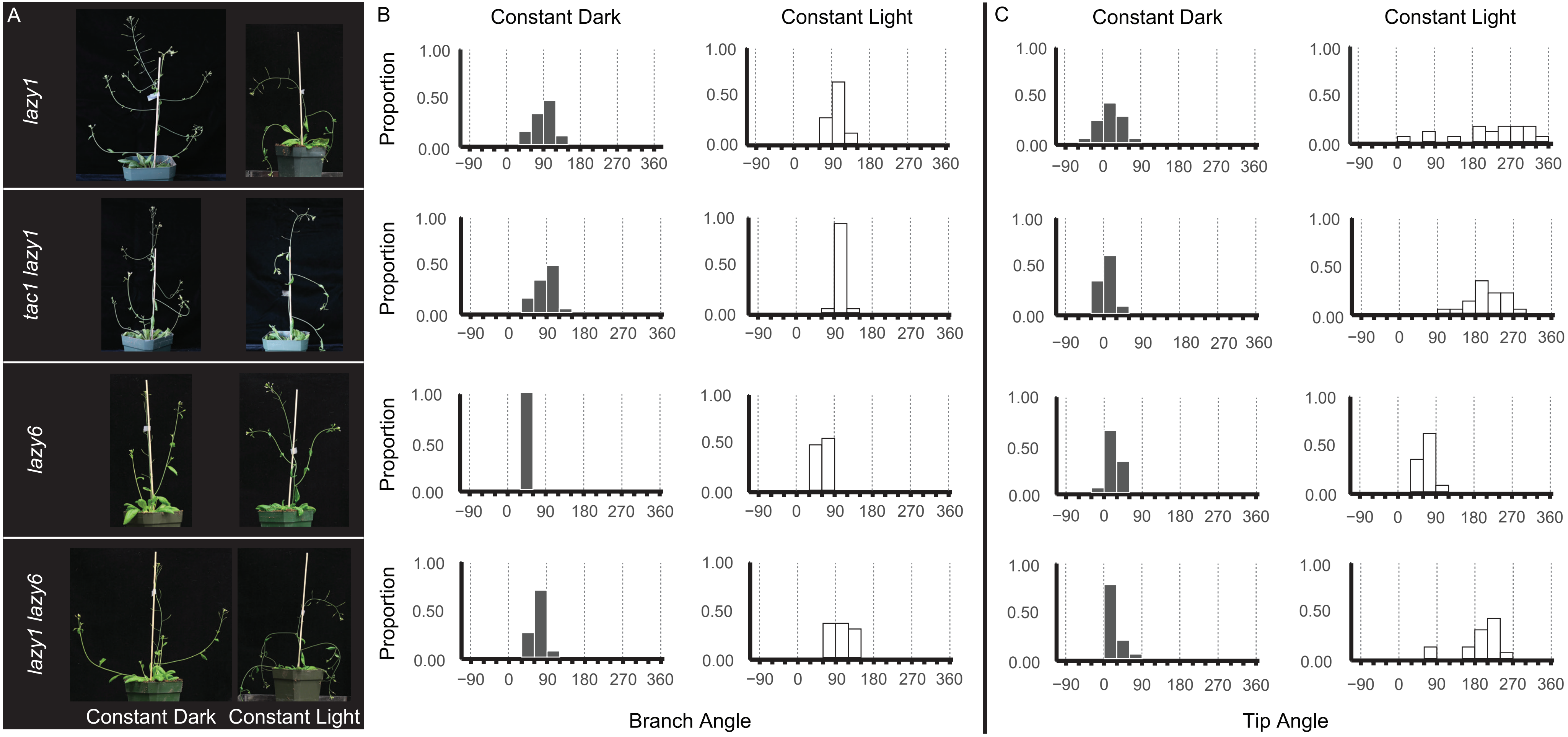

Figure S2. *tac1 lazy1* and *lazy1 lazy6* mutants largely phenocopy *lazy1* mutants in constant light and dark conditions.
A. Representative images of branch and tip angle phenotypes in *lazy1*, *tac1 lazy1*, *lazy6*, *lazy1 lazy6* mutants at the end of 72 hours of growth in constant light and dark conditions. B. Histograms of branch angles and tip angles that were quantified and binned into 30-degree bins. Values are reported as the proportion of total branches measured. For each genotype, n is as follows: *lazy1* - 16 branches from 6 plants (dark) and 16 branches from 5 plants (light); *tac1 lazy1* - 12 branches from 5 plants (dark) and 19 branches from 4 plants (light); *lazy6* - 22 branches from 5 plants (dark) and 15 branches from 5 plants (light); *lazy1 lazy6* - 21 branches from 5 plants (dark), and 17 branches from 5 plants (light). For scale, the width of the pots in both A and C is 86mm.

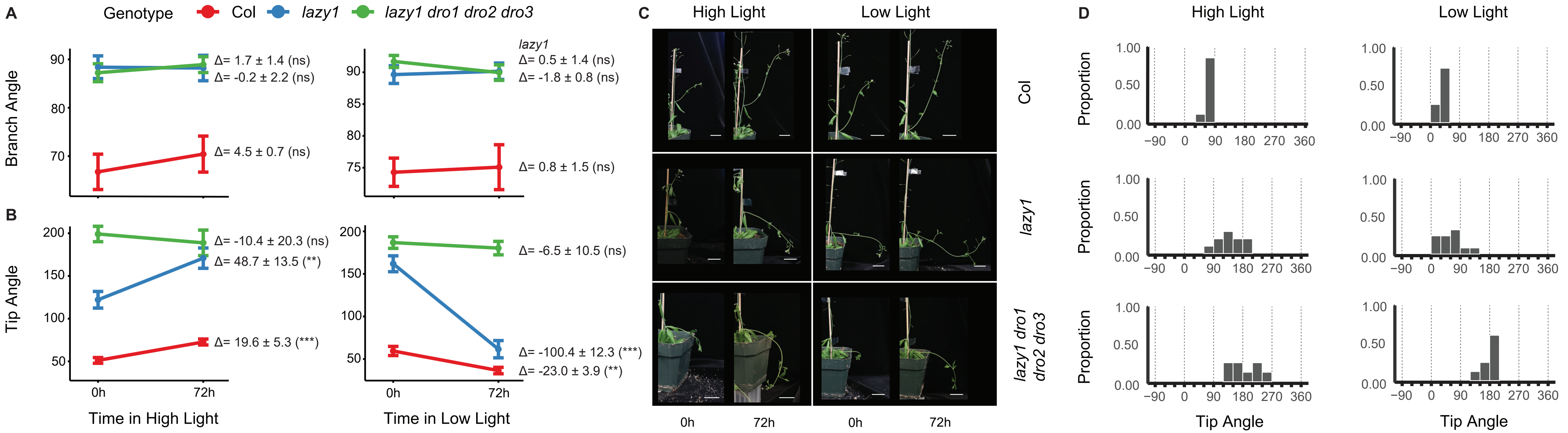

Figure S3. Quadruple *lazy/dro* mutant tip angles exhibit insensitivity to high and low light, while *lazy1* mutants appear hypersensitive.
A. Quantified branch angles (angle to 2cm from the branch point, with respect to the stem) and B. tip angles (tangent at 1 cm from the tipwith respect to the stem) for Col, *lazy1*, and *lazy1 dro1 dro2 dro3* quadruple mutants in high-light (left) and low-light (right) conditions. Error bars represent +/- standard error of the mean at each time point, with n's reported below. Angle change from 0 to 72 hours for individual branches was averaged and reported with +/- standard error of the mean to the right of each graph. A student's t-test was used to determine significant difference between 0 and 72 hour time points and is reported in parentheses. *p<0.05, **p<0.01, ***p<0.001. C. Representative initial and end-point images of Col, *lazy1*, and *lazy1 dro1 dro2 dro3* quadruple mutants in high-light (left) and low-light (right) conditions. White scale bars indicate 20mm in length. D. Histograms of branch angles and tip angles that were quantified and binned into 30-degree bins. Values are reported as the proportion of total branches measured. For each genotype, n is as follows: Col - 9 branches from 4 plants (high) and 8 branches from 4 plants (low); lazy1 - 10 branches from 4 plants (high) and 12 branches from 4 plants (low); quadruple - 8 branches from 4 plants (high) and 8 branches from 4 plants (low).

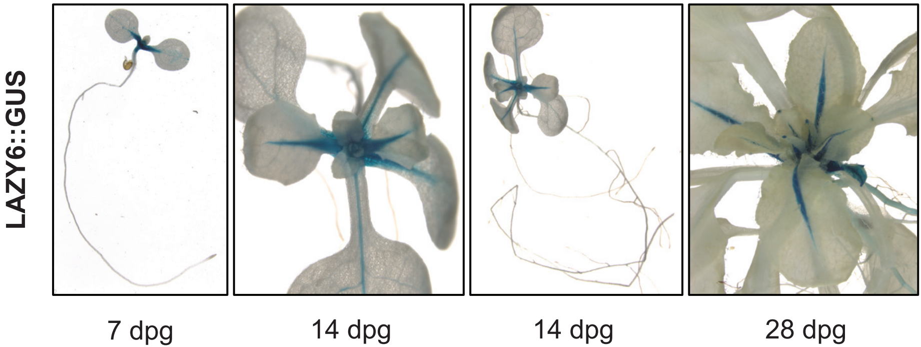

Figure S4. *LAZY6* is expressed in shoot vasculature through 28 dpg. pLAZY6::GUS is expressed in the upper hypocotyl and cotyledon petiole vasculature at 7 dpg, and continues to be most strongly expressed in leaf petiole vasculature and into leaf mid-veins, strongest in newly emerging leaves at 14 and 28 dpg. Representative lines are shown.

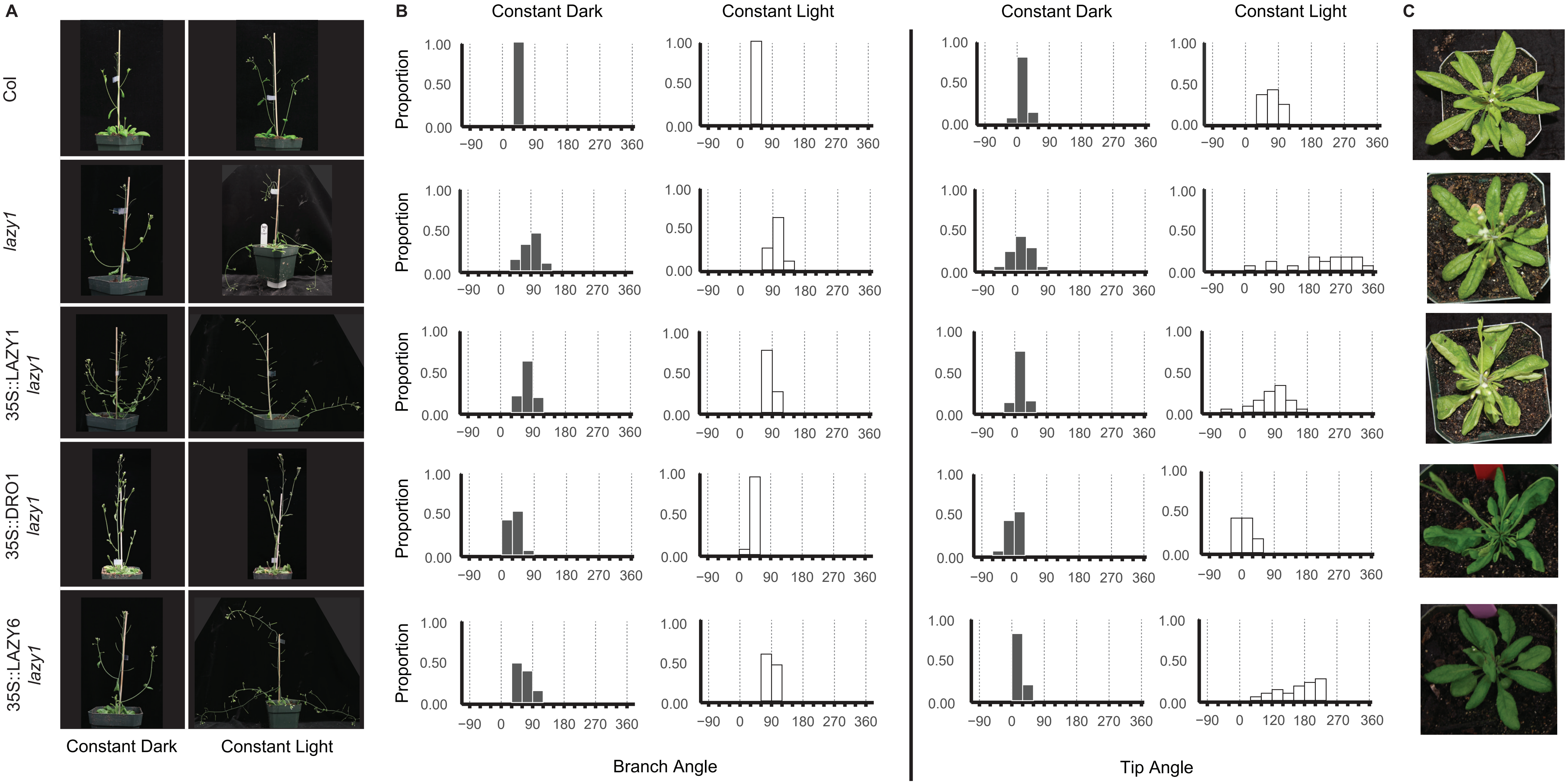

Figure S5. Overexpression of *LAZY1*, *DRO1*, and *LAZY6*, alters light-induced branch angle phenotypes in a *lazy1* mutant background.
A. Representative images of branch and tip angle phenotypes in Col, *lazy1*, and 35S::LAZY1, 35S::DRO1, and 35S::LAZY6 in a *lazy1* background at the end of 72 hours of growth in constant dark or constant light conditions. B. Histograms of branch angles and tip angles that were quantified and binned into 30-degree bins. Values are reported as the proportion of total branches measured. For reference, *lazy1* histograms are repeated here from Additional File 1: Fig. S2. For each genotype, n is as follows: Col - 15 branches from 5 plants (dark) and 17 branches from 5 plants (light); 35S::LAZY1 *lazy1* - 50 branches from 11 plants (dark) and 28 branches from 6 plants (light); 35S::DRO1 *lazy1* - 19 branches from 4 plants (dark) and 16 branches from 4 plants (light); 35S::LAZY6 *lazy1* - 21 branches from 4 plants (dark) and 23 branches from 4 plants (light). C. Representative images showing leaf curling phenotypes in 35S::LAZY1 *lazy1* and 35S::DRO1 *lazy1* genotypes, but not in others. For scale, the width of the pots in both A and C is 86mm.

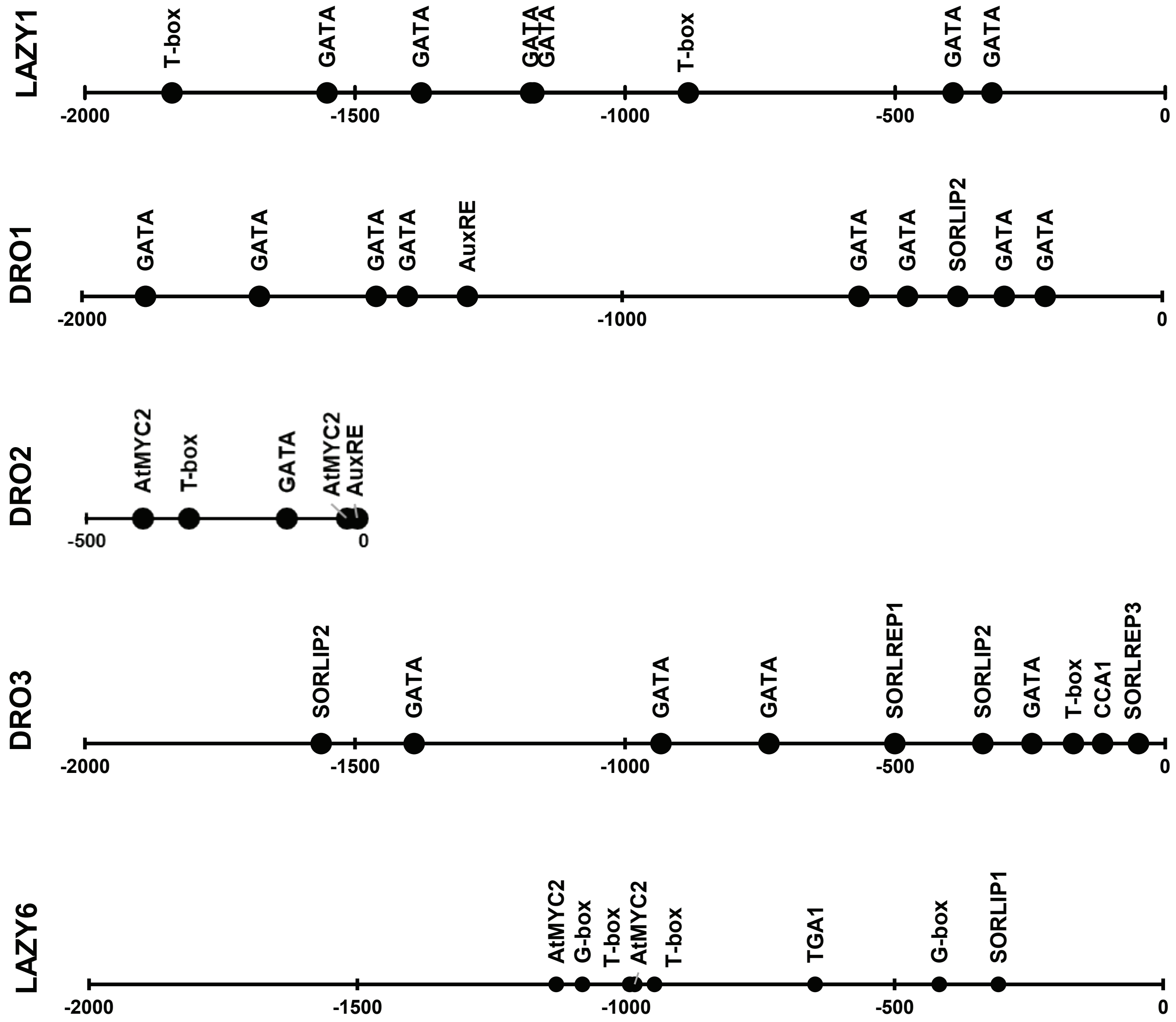

Figure S6: *LAZY* and *DRO* genes contain several types of light-related cis-elements. Using the AtCisDB database, we identified the light- and circadian-related cis-elements in the promoters of *LAZY* and *DRO* genes.

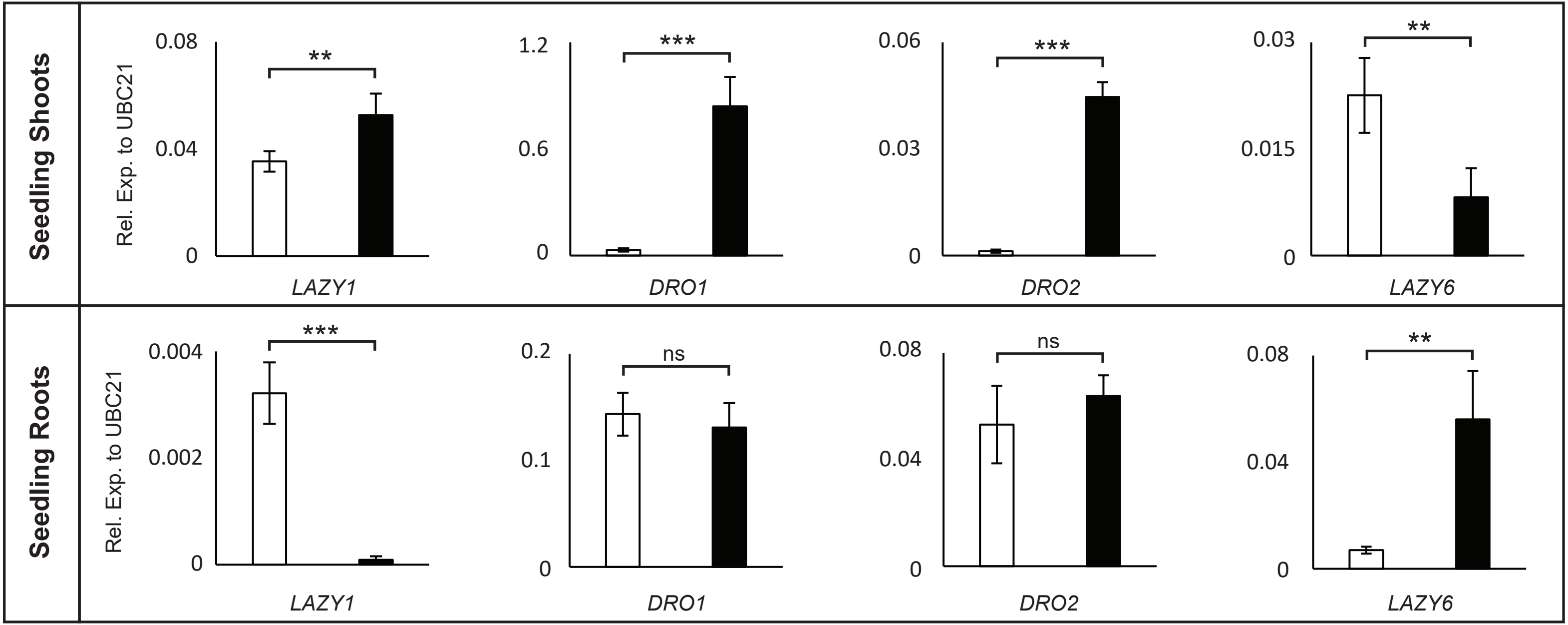

Figure S7. *LAZY* and *DRO* gene expression shows differing responses to light changes in seedling shoots and roots. Seedlings grown to 10d in 16L:8D light cycles were moved to continuous light or dark for 72 hours, and shoot and root tissues were collected separately. Expression relative to housekeeping gene *UBC21* is reported. Error bars represent SD between four biological replicates of 10-12 pooled seedlings (whole plate with 2 rows of 6 seedlings) per time point. A student's t-test was used to determine significant difference between continuous light and dark growth after 72 hours and is reported as asterisks above graphs. *p<0.05, **p<0.01, ***p<0.001.
